# COVID-19: Are Africa’s diagnostic challenges blunting response effectiveness?

**DOI:** 10.12688/aasopenres.13061.1

**Published:** 2020-04-17

**Authors:** Francis Kobia, Jesse Gitaka

**Affiliations:** 1 Research and Innovation, Mount Kenya University, Thika, 01000, Kenya; 2 Department of Biosciences, University of Milan - via Celoria 26, Milan, 20133, Italy; 3 Implementation Research, Centre for Resarch in Tropical Medicine and Community Development, Nairobi, 00100, Kenya

**Keywords:** Covid-19, Point-of-care diagnostics, Africa, response, effectiveness

## Abstract

Since its emergence in Wuhan, China in December 2019, novel Coronavirus disease - 2019 (COVID-19) has rapidly spread worldwide, achieving pandemic status on 11 ^th^ March, 2020. As of 1 ^st^ April 2020, COVID-19, which is caused by severe acute respiratory syndrome coronavirus 2 (SARS-CoV-2), had infected over 800,000 people and caused over 40,000 deaths in 205 countries and territories. COVID-19 has had its heaviest toll on Europe, United States and China. As of 1 ^st^ of April 2020, the number of confirmed COVID-19 cases in Africa was relatively low, with the highest number registered by South Africa, which had reported 1,380 confirmed cases. On the same date (also the date of this review), Africa had reported 5,999 confirmed cases, of which 3,838 (almost 65%) occurred in South Africa, Algeria, Egypt, Morocco and Tunisia, with the remaining 2,071 cases distributed unevenly across the other African countries. We speculate that while African nations are currently experiencing much lower rates of COVID-19 relative to other continents, their significantly lower testing rates may grossly underestimate incidence rates. Failure to grasp the true picture may mean crucial windows of opportunity shut unutilized, while limited resources are not deployed to maximum effect. In the absence of extensive testing data, an overestimation of spread may lead to disproportionate measures being taken, causing avoidable strain on livelihoods and economies. Here, based on the African situation, we discuss COVID-19 diagnostic challenges and how they may blunt responses.

## Emergence and global spread of SARS-CoV2

In December 2019, a spate of pneumonia cases of unknown cause was observed in Wuhan, Hubei province, China ( He *et al.*, 2020). Soon after, the causative agent for the novel illness was found to be a novel Coronavirus (2019-nCoV) ( Lu *et al.*, 2020; Zhou *et al.*, 2020; Zhu *et al.*, 2020). Coronaviruses (CoVs), are a large group of viruses that frequently cause mild respiratory disease in humans, including common cold ( Saif, 2004; NIAID, 2020). Hundreds of coronaviruses exist in wild and domestic animals. In the last 20 years, 3 highly infectious CoVs have crossed from animals into humans through spillover events and spread globally, causing severe respiratory illnesses ( Andersen *et al.*, 2020; Cui *et al.*, 2019). In November 2002, severe acute respiratory syndrome (SARS), a novel respiratory disease emerged in China and rapidly moved to other countries. Its causative agent was identified as a CoV and named SARS-CoV ( Drosten *et al.*, 2003; Fung & Liu, 2019). The SARS mini pandemic infected over 8,000 people and caused almost 800 deaths ( Cherry, 2004). In 2012, a novel respiratory disease, named Middle East respiratory syndrome (MERS), was identified in Saudi Arabia and the causative agent identified as MERS-CoV ( de Groot *et al.*, 2013; Zaki *et al.*, 2012). To date, MERS is estimated to have infected over 2,000 people and caused over 700 fatalities ( Ramadan & Shaib, 2019; WHO, 2017). In December 2019, a novel respiratory disease presenting with severe unexplained pneumonia emerged in Wuhan, Hubei province - China ( Huang *et al.*, 2020; Zhu *et al.*, 2020). Its causative agent was quickly identified as a novel CoV (2019-nCoV), which was named SARS-CoV-2 (severe acute respiratory syndrome coronavirus 2) ( Lu *et al.*, 2020; Zhou *et al.*, 2020). The disease caused by SARS-CoV-2 was named COVID-19 by the WHO ( WHO, 2020e). COVID-19 then rapidly spread within China, where it infected over 82,000 people and caused more than 3,000 fatalities, mainly in Hubei province ( WHO, 2020b). The disease’s spread accelerated globally, prompting the WHO to declare it a global pandemic on 11 ^th^ March 2020 ( Bedford *et al.*, 2020). As of 1 ^st^ April 2020, over 800,000 COVID-19 cases and more than 40,000 COVID-19-associated deaths had been confirmed in 205 countries and territories. Europe and North America are currently the continents most affected by COVID-19. So far, Africa has reported the lowest number of confirmed COVID-19 cases ( WHO, 2020b). As of writing, 5,999 COVID-19 cases have been reported in Africa with South Africa reporting the highest number. 5 countries (South Africa, Tunisia, Morocco, Egypt, and Algeria) account for close to 65% (3,838 cases) of the confirmed cases, with the remainder being unevenly distributed in the rest of the continent. Within the East African community, there have been a total of 222 confirmed cases ( African Arguments, 2020; WHO, 2020b).

## SARS-CoV-2 transmission and pathogenesis

SARS-CoV2, is an enveloped single stranded positive sense RNA virus belonging to the family *Coronaviridae* and genus Betacoronavirus ( Lai *et al.*, 2020). The SARS-CoV-2 virion ranges between 50-200nm in diameter and houses a 29,881 bp genome ( Chen *et al.*, 2020a; Chen *et al.*, 2020b). Among other genes, the SARS-CoV-2 genome encodes 4 structural proteins named spike (S), envelope (E), membrane (M) and nucleocapsid (N). The N protein holds the viral genome while S, M and E construct the viral envelope, where S mediates viral entry into the host cell ( Wu *et al.*, 2020). SARS-CoV-2 is easily transmissible. According to the WHO, the main mode of COVID-19 transmission is direct/indirect human-human contact, where the virus is transmitted in respiratory droplets or via contact routes. Droplet transmissions happen when one gets into close proximity, (typically within a meter) with an individual exhibiting respiratory symptoms, such as sneezing or coughing. Indirect transmission may occur when one touches objects handled by an infected individual and then touches their mouth, nose or eyes. Transmission has also been reported to occur via airborne droplet transmission. In such cases, the virus is contained in droplet nuclei, which are typically <5µm in diameter and can remain airborne for extended periods. Airborne transmission can occur over distances beyond 1 meter but such nuclei are typically generated by processes that generate aerosols, usually patient care procedures ( WHO, 2020d). As such, social distancing, rigorous hand washing, and avoiding touching the face have been recommended as means of minimizing transmission risk ( WHO, 2020a). Once SARS-CoV-2 has gained access to the host’s respiratory mucosa, it enters the host cells through an interaction between its S protein and the host cell’s ACE2 (angiotensin-converting enzyme 2) receptors ( Hoffmann *et al.*, 2020). Unlike other coronaviruses that cause upper respiratory tract disease only, SARS-CoV-2 is capable of colonizing the lower respiratory tract as well ( Heymann & Shindo, 2020). After infection, the virus incubates for a median period of about 5 days before the onset of symptoms and almost all infections become symptomatic by day 11 ( Lauer *et al.*, 2020; Rothan & Byrareddy, 2020). Symptoms include fever, fatigue, headache, dry cough, diarrhea and lymphopenia. While most patients experience mild symptoms that they overcome without need for hospital care, some experience serious complications including severe pneumonia, acute respiratory distress syndrome (ARDS), acute cardiac injury and acute ground glass opacity (GGO) that may necessitate life support ( Heymann & Shindo, 2020; Rothan & Byrareddy, 2020).

## COVID-19 diagnostic testing

COVID-19 diagnostic testing is recommended for individuals that satisfy the suspect case definition ( Leitmeyer *et al.*, 2020). According to the WHO organization, the decision to test should be based on clinical signs, epidemiological factors and the possibility of infection ( Leitmeyer *et al.*, 2020), such as contact with an infected individual. The WHO ( WHO, 2020c) defines a suspect case as one that:

a) Shows symptoms of acute respiratory illness i.e. fever and at least one respiratory disease symptom e.g. coughing and shortness of breath, and has travelled or resided in an area with community COVID-19 transmission in the 14 days prior to symptoms onset; or,
b) Shows acute symptoms of any respiratory illness and has been in contact with a confirmed or suspected case in the 14 days prior to the onset of symptoms; or,
c) Shows symptoms of acute respiratory illness i.e. fever and at least one respiratory disease symptom e.g. coughing and shortness of breath and requires hospitalization in the absence of alternative diagnosis that fully accounts for the symptoms.

Suspected cases should then be validated by laboratory tests. This is routinely done by carrying out nucleic acid amplification tests (NAAT). Currently, RT-PCR detection of unique sequences of the viral genome is the gold standard for COVID-19 testing where the N, E, S and RdRP (RNA-dependent RNA polymerase) genes are targeted. Sample handling should be carried out in a BSL-2 biosafety cabinet under strict adherence to personal protective equipment (PPE) guidelines. However, RT-PCR is labor intensive, severely constraining the capacity for quick turnaround times from sample collection to results transmission. In many contexts, getting results takes days ( NPR, 2020). As a consequence, laboratory testing of suspect cases is characterized by long wait periods and an exponential increase in demand for tests. To address this bottleneck, rapid diagnostic tests with turnaround times ranging between 10 and 30 minutes have been developed, even though most of these are currently undergoing clinical validation and therefore not in routine use ( ECDC, 2020).

## COVID-19 testing for surveillance and pandemic control

In addition to suspect case diagnosis, widespread COVID-19 testing is critical for disease monitoring and surveillance. Such testing is recommended so as to meet the following objectives ( WHO, 2020c):

a) Monitor disease trends in contexts of rapid person-person virus spread.
b) Quickly identify cases in countries/regions where the virus is not circulating.
c) Generate epidemiological data for risk assessment regionally, nationally or globally.
d) Generate epidemiological data to guide government responses in terms of policy, resource mobilization and distribution.

However, how extensively governments rollout testing for surveillance purposes is dependent on each country’s prevailing circumstances and preferred COVID-19 control strategy. For instance, aggressive, widespread testing in South Korea early in their epidemic, including the deployment of drive-through testing ( Kwon *et al.*, 2020) has been credited with effective spread control, avoiding the need for lockdown ( Colbourn, 2020). While in Italy, where medical services were rapidly overwhelmed by a high influx of severe COVID-19 cases, testing was restricted only to those with severe symptoms and needing hospitalization ( Onder *et al.*, 2020). This approach misses the vast majority of COVID-19 cases (estimated at around 80%) because they are mild or asymptomatic and tends to overestimate the disease’s fatality rate ( ISPI, 2020). This approach has also been blamed for contributing to the continued spread of COVID-19 as undiagnosed cases drive persistent community transmission ( Paterlini, 2020). Testing the entire population of the Italian village of Vo’Euganeo, and isolation of all positive cases has been reported to have helped eliminate COVID-19 from the village ( Day, 2020). Mass testing has been suggested as a means to quickly stop the COVID-19 epidemic in the UK ( Peto, 2020) and delayed roll out of large-scale testing is considered to have blinded the US to its worsening COVID-19 situation ( Cohen, 2020; New York Times, 2020). In Germany, large-scale testing has been credited for limiting disease spread and the low fatality rate reported by Germany relative to its neighbors ( Financial Times, 2020a). While many countries are ramping up surveillance testing, there are no guidelines for large-scale testing and decisions are based on individual countries’ assessments.

## Challenges of COVID-19 testing in African contexts

The benefits of large-scale COVID-19 testing have been demonstrated in several countries. However, most low-middle income countries (LMICs), including the majority of African countries, lack capacity for large scale testing. These countries are facing numerous challenges in their efforts to diagnose suspect cases, trace contacts for further testing and roll out surveillance testing. For instance, as a consequence of inadequate testing capacity, at the time of preparing this review Kenya had carried out 2,563 tests only, of which 122 returned positive ( MOH - Kenya, 2020). While Kenya’s ministry of health has indicated it is embarking on mass testing ( Xinhua, 2020), many challenges remain, including test kit shortages that have been reported in many parts of Africa ( VOA, 2020) as a result of high global demand.

A major diagnostic challenge stems from the nature of RT-PCR tests, the gold standard in COVID-19 testing. COVID-19 RT-PCR test kits are expensive, making the cost of large-scale testing prohibitive for LMICs. Additionally, the test requires expensive equipment, including PCR machines and adequately equipped BSL-2 labs. These factors, and the need for highly trained personnel, mean that in most African countries, very few centers have the capacity to run COVID-19 tests. Furthermore, completing a COVID-19 RT-PCR test is a multi-step process that begins with sample collection, often at a point of care that is far away from the country’s test center(s). The sample must then be carefully packaged and transported to the distant testing site under conditions that protect sample integrity. At the lab, laborious sample preparation, including RNA extraction is done, followed by reaction mix preparation and RT-PCR ( NPR, 2020). This multi-step, labor intensive process results in huge backlogs, severely slowing suspect case testing and limiting capacity for mass testing. While these challenges are global, countries with stronger research capacities have significantly higher test rates as private and academic research institutions help in testing ( Financial Times, 2020c). In fact, RT-PCR diagnostic capacity is severely limited in most African countries, with the number of qualified testing labs ranging between 1 and 3 in 40 African countries. To address this shortfall, the WHO-Afro and Africa Task Force for Coronavirus (AFTCOR) are keen to increase this number and bring testing capacity to all 55 African countries ( Nkengasong & Mankoula, 2020). Owing to these challenges, many LMICs governments must prioritize testing of suspect cases and traced contacts. In turn, inadequate testing severely occludes the proper understanding of prevalence and incidence of COVID-19, raising the risk of undetected community spread. Furthermore, a blinded view of COVID-19 means that government responses may not be commensurate with the actual situation. For instance, measures may be relatively weak where stronger measures would have been taken had the actual situation been captured. Likewise, measures may be too stringent in some contexts if identical risk is assumed countrywide in the absence of mass surveillance data. Hence, limited diagnostic capacity blinds governments’ COVID-19 assessments, limiting the country’s abilities to deploy limited resources to maximum effectiveness.

## Surmounting COVID-19 diagnostic challenges

 COVID-19 diagnostic challenges are not unique to African countries and LMICs. Consequently, numerous private and public institutions have developed rapid diagnostic tests (RDTs) aimed at speeding and expanding testing, crucial factors in the struggle to slow COVID-19 spread. RDTs, which are largely based on immunoassays, may be direct, through detection of SARS-CoV-2 antigens or indirect, through detection of anti-SARS-CoV-2 antibodies ( ECDC, 2020). Advantages of RDTs include ease of use as they do not require special equipment or highly trained personnel and stability at room temperature, removing the need for constant refrigeration/freezing. RDTs are therefore highly suited for point of care diagnosis (POCD) and are highly amenable to deployment in low resource settings, removing the need for sample transportation. Several COVID-19 RDTs, capable of giving results in 10-30 minutes are now commercially available or in development ( ECDC, 2020). In many African contexts, RDTs would reduce the time needed to get test results from days to minutes. Therefore, RDTs offer a means to aggressively deploy mass testing across Africa. However, the cost of RDTs for mass testing may still be prohibitively high, calling for homegrown solutions.

The COVID-19 diagnostic challenges faced by African nations highlight long-running diagnostic challenges for a wide range of diseases. Part of our group’s research has been the development of RDTs and POCDs for various diseases, including placental malaria and bacterial infections. We contend that an effective means of achieving mass testing at the required scale, is to fund the development COVID-19 RDTs locally, to meet local demand – bearing in mind that the knowledge and relevant local and international collaborations are already in place. Such solutions should then be aggressively deployed for points of care and home use, particularly in rural settings. In fact, this strategy is being used by Senegal, which together with UK collaborators, is developing an affordable COVID-19 RDT (expected to cost $1 per test) for home use in African countries ( Financial Times, 2020b). Similar approaches by other African countries would provide local solutions to the continent’s test needs while supporting Africa’s research and innovation.

## COVID-19 point of care testing strategies

Evidently, COVID-19 testing by RT-PCR is not applicable in most parts of Africa considering that vast populations live in rural settings with poor transport and communication infrastructure. RT-PCR requires expensive equipment, skilled personnel, reagents and reliable power supplies. Additionally, the long turnaround time of 4-24 hours (and days in some contexts) ( NPR, 2020), may discourage many from seeking tests. Thus, there is an urgent need for deployable, COVID-19 point of care tests satisfying the ASSURED requirements of an ideal diagnostic test. Meaning that such tests should be Affordable, Sensitive, Specific, User-friendly, Rapid, Equipment-free, and Delivered to those in need ( Mabey *et al.*, 2004; Urdea *et al.*, 2006). Efficient and accurate testing will enable early diagnosis of Covid-19 for timely clinical care and tracing of contacts for isolation and quarantine measures. The need for prompt reliable testing is made dire by findings that COVID-19 positive people with mild or no symptoms may actually harbor high viral loads in the throat and may transmit it by ‘viral shedding’ ( Woelfel *et al.*, 2020).

Different strategies exist for point of care testing, all with inherent merits and demerits: 1) Use of antibody testing. As the body mounts an immune response, antibodies against SARS-CoV-2 antigens are generated. These antibodies may serve as indicators of infection. However, since detectable antibodies may lag behind the appearance of clinical symptoms, most infections will be missed, causing a high rate of false negatives. Conversely, persistence of antibodies after virus elimination from the body may result in a high rate of false positives. Moreover, a high cross reactivity of SARS-CoV-2 and SARS-CoV S protein against plasma samples from 15 patients has been observed, which may impact test interpretation ( Lv *et al.*, 2020). However, in spite of these drawbacks, antibody tests will still be useful in community surveillance of exposed populations and this information will be useful in determining the extent of ‘herd’ immunity and guide tailored public health interventions. 2) Antigen point of care tests overcome the problems inherent in antibody tests by showing evidence of viral proteins, thereby indicating ongoing virus infection. However, such tests will not be as sensitive as to RT-PCR and their clinical utility is yet to be determined, especially in the context of other CoVs. Several of these tests, for example Spike Dart from E25Bio ( The Boston Globe, 2020), are currently under evaluation for FDA approval. In Europe, antibody and antigen tests are now commercially available ( ECDC, 2020). 3) Molecular based testing based on isothermal amplification such as RT-LAMP and CRISPR based SHERLOCK (Specific High Sensitivity Enzymatic Reporter UnLOCKing) ( Kellner *et al.*, 2019; Zhang *et al.*, 2020) have the potential for bridging the gaps left by antibody based tests but pose unique challenges for field deployment. These require heating, reagents that may need a cold chain and some degree of sample preparation. Past experiences with LAMP, which has been used for malaria, TB and several other infectious diseases demonstrate that important implementation bottlenecks need to be overcome to move these from research settings to routine use. The GeneXpert platform based Xpert® Xpress SARS-CoV-2 test by Cepheid, has received emergency use approval by the FDA ( Cepheid, 2020). Fortunately, this platform has widespread use for TB diagnosis in Africa and repurposing it for COVID-19 will fast track testing. However, supply chain management challenges previously experienced during TB testing will need to be overcome for meaningful operation.

Other near point of care tests include radiological imaging with Chest CT-scan. This approach has been shown to be highly sensitive (at 97%) but poorly specific (at 48%) in a Chinese study ( Ai *et al.*, 2020). CT-Scan, unlike RT-PCR enables shorter result times especially when coupled with artificial intelligence (AI) enabled image analysis and interpretation. But this technology is highly limited to higher level hospitals in most African countries, with very low numbers of radiologists and poor adoption of AI. This technology may not be sufficient in addressing the COVID-19 diagnosis challenges in Africa.

Given these considerations, it is clear that the current gold standard tools, RT-PCR and radiological imaging, cannot adequately meet Africa’s COVID-19 diagnosis challenges in the low resource settings that characterize most hospitals in sub-Saharan Africa. Indeed, as the pandemic situation evolves with control goals focusing on both containment and mitigation ( Parodi & Liu, 2020; WHO, 2020f), capacity for large-scale diagnosis at most if not all levels of health care systems will be vital for sustainable control. Crucially, as treatment solutions for the disease become available, prompt diagnosis will be essential in ensuring prompt treatment and determining isolation/quarantine decisions.

## Conclusion

COVID-19 has severely tested the adequacy of global diagnostic preparedness and ability to rapidly develop point of care tests for emerging infections. The prompt release of SARS-Cov-2 whole genomic sequence data by Chinese scientists helped with development of RT-PCR protocols that have been used worldwide. However, as the pandemic evolves, it is increasingly important to develop point of care tests that will facilitate proper last mile epidemiology, inform treatment and public health interventions. These POC tests will leverage available molecular platforms such as CRISPR, or be based on antigen or antibody detection. Critically, it should be understood that these strategies have inherent merits and demerits and synergy will only be achieved where all are used appropriately. Additionally, the COVID-19 pandemic has highlighted the need for development and growth of in-continent POC diagnostics development capacity ranging from assay development, device fabrication, prototyping, validation, implementation research and entrepreneurial ecosystems including venture capitalization and regulation.

## Data availability

### Underlying data

No data are associated with this article.
