# Supplementary material for: COVID-19: Are Africa’s diagnostic challenges blunting response effectiveness?
Source: AAS Open Res. 2020 Apr 17;3:4. [Version 1] doi: 10.12688/aasopenres.13061.1 (PMC7205356; doi:10.12688/aasopenres.13061.1)
Supplement: Peer review history

# Peer review: Reviewer response for version 1

**DOI:** 10.21956/aasopenres.14156.r27421

**Published:** 2020-05-05

**Authors:** Jacqueline C. Linnes

**Affiliations:** 1 Purdue University, West Lafayette, IN, USA

The authors provide a concise and thorough review covering COVID-19 emergence, pathogenicity, transmission and diagnostic methods, as well as challenges faced by African countries. The authors indicate that current gold standard testing methods such as RT-PCR and Chest CT scans are not accessible options given the long turnaround time (RT-PCR) and limited available personnel and implementation for these technologies. The authors describe the need for funding local development and production of RDTs to detect COVID-19 in order to achieve widespread availability and testing. The present form of the manuscript should be published given its valuable contributions for scientific review and policy but could be further improved with minor changes described below:

 In section “SARS-CoV-2 transmission and pathogenesis”, the authors indicate that “…almost all infections become symptomatic by day 11 (Lauer *et al.*, 2020; Rothan & Byrareddy, 2020).” However, current research (after this review was submitted, e.g. Arons *et al.*, 2020 ^1^) indicates that many cases are asymptomatic. It would be better to qualify the statement so as not to imply that asymptomatic infection is rare.

 In the COVID-19 point of care testing strategies, it is possible that a confirmatory testing protocol for RDTs could be implemented if specificity is insufficient. This could be done with with a second RDT against different antibody reactivities the way that HIV test protocols are commonly performed. Even in the case of imperfect tests, extensive testing is better than none at all.

 The conclusion should definitively answer the title question: Are Africa’s diagnostic challenges blunting response effectiveness? The authors have described diagnostic challenges and how they have limited effectiveness in other situations, but have not answered whether the diagnostic challenges specifically are currently limiting response effectiveness in Africa.

 The conclusion indicates need for increased capacity for "assay development, device fabrication, prototyping, validation, implementation research and entrepreneurial ecosystems" (which I agree with) but the review does not highlight how this might be done and could benefit from examples of success overcoming these challenges in the diagnostics or other fields. 

 The “ECDC: An overview of the rapid test situation for COVID-19 diagnosis in the EU/EEA. 2020” link currently goes to the wrong location but should go to the website: https://www.ecdc.europa.eu/en/publications-data/overview-rapid-test-situation-covid-19-diagnosis-eueea or the PDF itself: https://www.ecdc.europa.eu/sites/default/files/documents/Overview-rapid-test-situation-for-COVID-19-diagnosis-EU-EEA.pdf

Is the review written in accessible language?

Yes

Are all factual statements correct and adequately supported by citations?

Yes

Are the conclusions drawn appropriate in the context of the current research literature?

Yes

Is the topic of the review discussed comprehensively in the context of the current literature?

Yes

Reviewer Expertise:

development of point-of-care diagnostics for infectious diseases, microfluidics, biosensors,

I confirm that I have read this submission and believe that I have an appropriate level of expertise to confirm that it is of an acceptable scientific standard.

## References

[1] : Presymptomatic SARS-CoV-2 Infections and Transmission in a Skilled Nursing Facility. *N Engl J Med*.2020; 10.1056/NEJMoa2008457 10.1056/NEJMoa2008457 32329971PMC7200056

# Peer review: Reviewer response for version 1

**DOI:** 10.21956/aasopenres.14156.r27422

**Published:** 2020-05-04

**Authors:** Kennedy O. Okeyo

**Affiliations:** 1 Institute for Frontier Life and Medical Sciences, Kyoto University, Kyoto, Japan

The present review by the authors provides important information on diagnostic challenges facing African countries in their combat against the ongoing COVID-19 pandemic. The paper reviews the history of the COVID-19 and traces its spread to Africa, details the mechanisms of transmission and pathogenesis of the disease in a concise and easy-to-understand style, and provides an in-depth examination of the diagnostic challenges Africa faces in assessing the full scale of the pandemic in the continent. The authors contend that most African countries lack the capacity to administer mass screening to ascertain the extent of the disease spread, and call for support toward the development of homegrown RDTs and POCTs as a strategy to achieve mass screening of COVID-19 in Africa.

 Overall, the paper succeeds in presenting a concise review of COVID-19 situation in Africa, identifies diagnostic challenges and suggests POCTs as a widely deployable testing approach which, if implemented for mass screening, can be effective for monitoring the spread of the ongoing pandemic.

 Hence, I find the paper to be of reasonable priority in view of the current situation, and recommend its publication even in the present form. I believe that paper contains scientifically valuable information and would be an important resource for scientists as well as policy makers in the battle against the ongoing scourge not only in Africa but worldwide.

 However, in revising the article for publication, the authors may wish to consider the following minor comments/corrections:

1. The authors ought to mention the impact of mass screening/diagnostics on the existing weak health infrastructure in Africa. The success story from South Korea arose out of a highly coordinated system of diagnosis, isolation and treatment, but this may not be possible with the underdeveloped healthcare infrastructure in Africa. One concern would be that identifying positives without appropriate follow up measures can lead to public fear and stigmatization, which might undermine the intended purpose of mass screening.
2. Poor research funding has always been cited as a major hindrance to the development of homegrown scientific solutions in Africa. The authors ought to comment on the challenges facing the suggested development of RDT/POC in Africa, most notably, lack of financial support and political goodwill. Specific to the present COVID-19 case, would it be faster and cheaper importing the diagnostic tools, as is already being done by some countries?
3. The authors may wish to put “ **COVID-19 point of care testing strategies**” section before “ **Surmounting COVID-19 diagnostic challenges**” section, for consistency with the conclusion.
4. The authors ought to provide a clear-cut answer to the question **“Are Africa’s diagnostic challenges blunting response effectiveness?”** which appears in the title. It would be nice to reassess and address the question by providing an answer to it in the conclusion part.
5. It would be informative to provide a list of some of the RDTs/POCTs available for rapid deployment or, alternatively, cite this paper (access via the URL below) which provides an updated list of POCTs.  
https://aasopenresearch.org/articles/3-8/v1
6. The link for the reference below is not correct. Please check.

 

 Ref: ECDC: An overview of the rapid test situation for COVID-19 diagnosis in the EU/EEA. 2020.

 The link provided in the manuscript opens up this page:

https://www.medrxiv.org/content/10.1101/2020.03.18.20038059v1

Is the review written in accessible language?

Yes

Are all factual statements correct and adequately supported by citations?

Yes

Are the conclusions drawn appropriate in the context of the current research literature?

Yes

Is the topic of the review discussed comprehensively in the context of the current literature?

Yes

Reviewer Expertise:

Bioengineering, Biotechnology, Biomechanics, Microfluidics

I confirm that I have read this submission and believe that I have an appropriate level of expertise to confirm that it is of an acceptable scientific standard.
